# Dietary Nonadherence and Its Determinants Among Diabetes Mellitus Patients in Comprehensive Specialized Hospitals, Amhara Region, Ethiopia: A Cross‐Sectional Study

**DOI:** 10.1002/hsr2.71154

**Published:** 2025-08-11

**Authors:** Makda Abate Belew, Rediet Akele Getu, Sewunnet Azezew Getahun, Belete Negese Gemeda, Melese Wagaye Zergaw, Sewnet Getaye Workie

**Affiliations:** ^1^ Department of Nursing, School of Nursing and Midwifery, Asrat Woldeyes Health Science Campus Debre Berhan University Debre Berhan Ethiopia; ^2^ Department of Midwifery, School of Nursing and Midwifery, Asrat Woldeyes Health Science Campus Debre Berhan University Debre Berhan Ethiopia; ^3^ Department of Epidemiology and Biostatistics, School of Public Health, Asrat Woldeyes Health Science Campus Debre Berhan University Debre Berhan Ethiopia

**Keywords:** adherence, diabetes mellitus, diet, Ethiopia, nonadherence

## Abstract

**Background and Aim:**

Dietary nonadherence remains a significant challenge in diabetes management, compromising glycemic control and increasing the risk of complications. In Ethiopia, particularly in the Amhara region, adherence to dietary recommendations is influenced by cultural, economic, and educational factors. Despite the critical role of diet in diabetes management, there is limited evidence regarding dietary nonadherence and its determinants in this setting. This study assessed the prevalence and factors associated with dietary nonadherence among diabetes patients attending follow‐up care in the Amhara region comprehensive specialized hospitals of Ethiopia.

**Method and Materials:**

A multistage sampling technique was used to conduct an institution‐based cross‐sectional study among 497 diabetes mellitus patients attending follow‐up care at Debre Berhan, Debre Markos, Felege Hiwot, and Dessie Comprehensive Specialized Hospitals. The Perceived Dietary Adherence Questionnaire (PDAQ) was used to assess Dietary adherence. Multivariable binary logistic regression analysis was conducted to identify determinants of dietary nonadherence, and findings were reported using adjusted odds ratios (AOR) with 95% confidence intervals (CI).

**Results:**

Among the 497 individuals studied (mean age: 56.2 ± 12.3 years), 41.6% (95% CI: 37.4–46.0) failed to adhere to dietary recommendations. The identified factors were being single (AOR = 2.518; 95% CI: 1.248–5.083), being a farmer (AOR = 5.032; 95% CI: 1.772–14.293), rural residence (AOR = 0.307; 95% CI: 0.144–0.658), low family income (AOR = 3.707; 95% CI: 1.623–8.465), poor social support (AOR = 2.535; 95% CI: 1.456–4.412), and moderate social support (AOR = 1.861; 95% CI: 1.118–3.097).

**Conclusion:**

A high level of dietary nonadherence was observed among diabetic patients. To enhance dietary adherence, targeted nutritional education, community health promotion, and improved social support systems are recommended, especially in rural areas.

**Abbreviations**
ADA: American Diabetes Association
AOR: adjusted odds ratio
BMI: body mass index
CI: confidence interval
COR: crude odd ratio
DM: diabetes mellitus
HC: hip circumference
OSLO: Oslo Social Support Questionnaire
PDAQ: Perceived Dietary Adherence Questionnaire
WC: waist circumference
WHR: waist to hip ratio

## 1 Introduction

Diabetes mellitus (DM) is a chronic metabolic disorder characterized by persistent hyperglycemia due to inadequate insulin secretion, insulin resistance, or a combination of both pathophysiological mechanisms [1]. The International Diabetes Federation (IDF) reported that approximately 589 million adults aged 20–79 years were living with diabetes globally in 2024, with projections of 853 million by 2050 [2]. The burden of diabetes is increasing significantly in sub‐Saharan Africa, where Ethiopia ranks fifth in the number of affected adults [2]. In Ethiopia, the number of adults living with diabetes has risen from about 268,100 in 2000 to an estimated 2.3 million in 2024, with projections indicating a rise to 6.0 million by 2050 [2]. A systematic review and meta‐analysis conducted in Ethiopia reported a pooled national prevalence of diabetes mellitus of 6.26% based on 15 studies, highlighting the growing concern of diabetes in the region [3]. Globally, diabetes and its complications were responsible for approximately 3.4 million deaths among adults aged 20–79 years in 2024, emphasizing its significant impact on public health [4].

Effective diabetes management requires balancing healthy medication, exercise, and dietary modifications. Dietary recommendations emphasize the consumption of whole grains, fruits, vegetables, legumes, and nuts while limiting alcohol intake and reducing consumption of refined grains, red and processed meats, and sugary beverages. Furthermore, they emphasize the intake of less fat, less sodium, more fiber, and more foods such as fish and soya products with health‐promoting properties [5, 6].

The goals of nutrition therapy for individuals with diabetes have evolved to become more flexible and patient‐centered. According to the American Diabetes Association (ADA) 2019 report, these goals focus on promoting and supporting healthy eating patterns that include a variety of nutrient‐dense foods in appropriate portion sizes to enhance overall health. This includes achieving and maintaining a healthy body weight, meeting personalized blood glucose, blood pressure, and lipid levels targets, and preventing or delaying diabetes‐related complications. The approach also emphasizes preserving the enjoyment of eating by offering nonjudgmental guidance on food choices. Rather than concentrating on specific macronutrients, micronutrients, or individual foods, the focus is on equipping individuals with practical tools for everyday meal planning. Additionally, nutrition therapy is tailored to address each person's unique needs, considering cultural and personal preferences, health literacy and numeracy, access to healthy foods, readiness and ability to make behavioral changes, and any barriers they may face [5, 6].

Various factors across different levels may impact adherence to recommended dietary guidelines. At the individual level, factors such as motivation, personal knowledge, perceptions regarding moderation, self‐responsibility, taste preferences, and temptations play a significant role. Within small groups, including family and friends, relationships are identified as crucial support systems for managing diabetes. At the organizational or health systems level, challenges such as prolonged waiting times and the need to consult multiple doctors can negatively impact patient compliance. At the community and policy levels, cultural influences and food affordability influence adherence to dietary recommendations [7].

Factors leading to dietary nonadherence have been identified in different countries of the world, including Ethiopia [7, 8, 9, 10, 11, 12, 13, 14, 15, 16, 17, 18, 19]. Educational barriers, including low levels of education and insufficient dietary knowledge, impede individuals from making informed choices about their diets. Health‐related issues, such as the presence of comorbidities, a short duration of diabetes, and mental health challenges like depression, further complicate adherence to dietary guidelines. Social and economic influences, including poor support networks and low income, restrict access to healthy food options. Additionally, individuals often struggle with food choices, particularly during social gatherings, and face challenges due to the unavailability of fruits and vegetables. Finally, occupational factors also affect dietary habits by influencing both food access and the time available for meal preparation. Addressing these interconnected issues is crucial for enhancing dietary adherence in these regions [7, 8, 9, 10, 11, 12, 13, 14, 15, 16, 17, 18, 19].

Despite the significance of adhering to dietary recommendations for managing DM [13], the prevalence of dietary nonadherence among individuals with DM remains substantial, with rates ranging from 24% to 87.5% globally [10, 20, 21], 24% to 37% in the continent of Africa [5, 22, 23], and 46.8% to 74.3% in Ethiopia [11, 15, 17, 18, 19, 24]. Furthermore, there is limited concrete evidence regarding the magnitude and determinants of dietary nonadherence among individuals with diabetes in Ethiopia, particularly in the Amhara region. Therefore, this study aimed to assess the prevalence and factors associated with dietary nonadherence among diabetes patients attending follow‐up care in the Amhara region comprehensive specialized hospitals of Ethiopia.

## 2 Materials and Methods

### 2.1 Study Setting, Design, and Study Period

An institution‐based cross‐sectional study was conducted between November 17, 2022, and December 18, 2022, among patients with diabetes mellitus attending follow‐up clinics at selected comprehensive specialized hospitals in the Amhara region. The region has eight comprehensive specialized hospitals, with approximately 18,573 diabetic patients receiving follow‐up care.

Four hospitals—Debre Berhan, Debre Markos, Dessie, and Felege Hiwot—were randomly selected for the study. These hospitals operate chronic disease follow‐up clinics on all working days, each serving an average of 25–30 diabetic patients daily.

### 2.2 Population

All adult patients with diabetes mellitus attending chronic follow‐up clinics at comprehensive specialized hospitals in the Amhara region were the source population. The study population included all adult diabetes patients attending the chronic disease follow‐up clinics at the selected hospitals during the study period. Patients who were critically ill or had severe medical conditions that prevented them from completing the study questionnaire in a single session, as well as newly diagnosed diabetes patients who had not been on regular diabetes medication for at least 6 months, were excluded from the study.

### 2.3 Sample Size Determination and Sampling Procedure

The study's sample size was calculated using a single population proportion formula, with the prevalence of dietary nonadherence (P) set at 74.3% [15] and a 95% confidence level along with a 5% margin of error. Following adjustments for a 10% anticipated nonresponse rate and the application of a design effect of 1.5 to address sampling variability, the final sample size was determined to be 503 participants.

**Figure 1:**
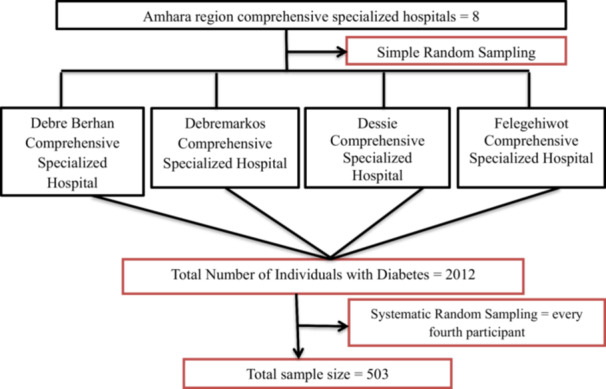
Diagrammatic presentation of the sampling procedure for assessing the level of dietary nonadherence and its associated factors among diabetes patients in selected comprehensive specialized hospitals of the Amhara region.

A multistage sampling method was employed to select participants for this study (Figure 1). The Amhara region has a total of eight comprehensive specialized hospitals, from which four (Debre Berhan, Debre Markos, Dessie, and Felege Hiwot) were selected through simple random sampling to serve as study sites. Next, systematic random sampling was used to choose eligible study participants from the pool of individuals with diabetes who had follow‐up appointments during the data collection period, as recorded in the hospitals' registration books. The sampling interval (*K*) was determined using the formula *K* = *N*/*n*, where *N* represents the total number of diabetic patients with follow‐up appointments at the four selected hospitals during the data collection period (2012), and *n* denotes the final sample size (503). Based on this calculation, every fourth patient was selected for the interview until the target sample size was achieved. The initial participant was chosen randomly using a lottery method [25].

### 2.4 Study Variables

The dependent variable in this study was dietary nonadherence among patients with diabetes mellitus, while the independent variables comprised sociodemographic, behavioral, psychosocial, and clinical‐related factors.

### 2.5 Data Collection Tools and Procedures

Data collection tools for this study were developed based on a comprehensive review of relevant literature. Instruments were validated to ensure accuracy and relevance in assessing dietary adherence among individuals with diabetes [26, 27, 28, 29, 30, 31, 32, 33]. Data was collected by six BSc nurses and overseen by three MSc students. The questionnaire contains five parts: sociodemographic data, perceived dietary adherence questions, clinical factors, behavioral and psychosocial factors, anthropometric measurements, and a data extraction checklist.

The PDAQ, a nine‐item assessment tool, is used to evaluate the level of dietary nonadherence. Respondents rate their adherence over the last 7 days on a 7‐point Likert scale, answering questions such as “On how many of the last 7 days did you…?” Higher scores generally indicate better dietary adherence, with the exception of items 4 and 9, which are reverse‐scored as they pertain to unhealthy food choices, such as foods high in sugar or fat. For these items, higher scores indicate poorer adherence. Therefore, their scores are reversed when calculating the total PDAQ score. Patients are classified as having good dietary adherence if they follow a healthy diet for at least 4 days per week and as having poor dietary adherence if they do so for fewer than 4 days per week [34]. This tool has been used in studies conducted in Ethiopia to assess dietary adherence among patients with diabetes mellitus [15, 35, 36].

The level of social support was assessed using the Oslo 3 Social Support Scale (OSS‐3), which asks patients to rate the support they receive from family and friends. This tool has been validated in various African countries [37] and utilized in studies conducted in Ethiopia [38]. The OSS‐3 score ranges from 3 to 14, with scores categorized as follows: 3–8 indicates “poor social support,” 9–11 indicates “moderate social support,” and 12–14 indicates “strong social support”.

Recent clinical and biochemical data, including fasting blood glucose levels, presence of comorbidities, diabetes‐related complications, duration of diabetes, and treatment methods, were collected from patients' medical records using standardized checklists. Participants were considered to have achieved adequate glycemic control if the average of their last three fasting blood glucose measurements was between 70 and 130 mg/dL. Those with average fasting blood glucose above 130 mg/dL were classified as having inadequate glycemic control [39].

### 2.6 Anthropometric Measurements

After participants stood with their arms at their sides, feet close together, and weight evenly distributed, waist circumference (WC) was measured to the nearest 0.1 cm at the end of a normal exhalation, with the measuring tape positioned at the level of the iliac crest. Hip circumference was measured to the nearest centimeter at the widest part of the buttocks. Waist circumference was considered above normal if it exceeded 94 cm for males and 80 cm for females [40, 41]. Both hip and waist circumferences were measured using a stretch‐resistant tape wrapped snugly around the participants [40, 41]. The waist‐to‐hip ratio (WHR) was calculated by dividing waist circumference (cm) by hip circumference (cm). A WHR of ≥ 0.90 for males and ≥ 0.85 for females was considered above normal [40].

Participants' heights were measured to the nearest centimeter using a stadiometer while standing upright. Weight was measured in light clothing using a calibrated Seca digital weighing scale (model 874, manufactured by Seca GmbH & Co., KG, Hamburg, Germany) and recorded to the nearest 0.1 kg. Body mass index (BMI) was computed by dividing weight in kilograms by height in meters squared (kg/m²) [40]. The scale was regularly calibrated, and the zero reading was checked before each measurement. Participants were classified as underweight if their BMI was less than 18.5 kg/m², healthy weight if their BMI ranged from 18.5 to 24.9 kg/m², overweight if their BMI was between 25 and 29.9 kg/m², and obese if their BMI was 30 kg/m² or higher [42].

### 2.7 Data Quality Assurance

The questionnaire was initially developed in English, translated into Amharic, and then back‐translated into English by independent language experts to ensure consistency. Before data collection, the instrument was pretested on 5% of the study sample (25 participants) at Debre Tabor Comprehensive Specialized Hospital to evaluate its simplicity, clarity, and ease of understanding. The principal investigator provided a 1‐day training session on interview techniques and measurement procedures to six data collectors and three supervisors. Quality control measures included random assessments of completed questionnaires at the end of data collection and thorough checks during data processing to ensure completeness and accuracy of the information.

### 2.8 Data Processing and Analysis

Data completeness was ensured by assigning a unique identifier to each questionnaire. Data entry was performed using EpiData version 3.1 [43], and statistical analyses were conducted using IBM SPSS Statistics version 21 [44]. The data were checked through visualization, frequency calculation, and sorting. Bivariate logistic regression analyses evaluated associations between each independent variable and the outcome. To adjust for potential confounders, the multivariable logistic regression model included variables with a *p* value < 0.25 in bivariate analyses. The multivariable model was pre‐specified to identify independent predictors of the outcome. Variables with a *p* value < 0.05 were considered statistically significant. Adjusted odds ratios (AORs) with 95% confidence intervals (CIs) were reported to quantify the strength and precision of associations. Model fitness was evaluated using the Hosmer–Lemeshow goodness‐of‐fit test [45], with a *p* value of 0.361 indicating an adequate model fit. All statistical tests were two‐sided, with a significance level (α) set at 0.05.

### 2.9 Ethical Approval and Consent to Participate

Ethical clearance for this study was obtained from the Institutional Review Board of Bahir Dar University, College of Medicine and Health Sciences, in accordance with national and institutional ethical guidelines. A formal letter of cooperation was submitted to the selected hospitals. Before the data were collected, written informed consent was obtained from all participants, and confidentiality was strictly maintained throughout the study.

## 3 Result

### 3.1 Sociodemographic Characteristics

Four hundred ninety‐seven individuals with diabetes mellitus participated in this study, resulting in a response rate of 98.3%. Of the total respondents, 50.9% were male. The study participants' mean age (± standard deviation) was 56.2 (±12.283). Occupation‐wise, 109 (21.9%) were government employees, and 71.6% were married. Of all participants enrolled in this study, 210 (42.3%) had an average monthly income of > 3000 ETB (Table 1).

**Table 1:** Sociodemographic characteristics of study participants with diabetes mellitus attending chronic follow‐up clinics in selected comprehensive specialized hospitals of the Amhara region.

Variables	Category	Frequency (*n* = 497)	Percent (100%)
Sex of the patient	Male	253	50.9
	Female	244	49.1
Age	≤ 50	174	35.0
	51–60	141	28.4
	61–70	123	24.7
	> 70	59	11.9
Marital status	Married	356	71.6
	Single	47	9.5
	Divorced	43	8.7
	Widowed	51	10.3
Education status	Unable to read and write	110	22.1
	Read and write	69	13.9
	Primary school	88	17.7
	Secondary school	53	10.7
	Preparatory school	71	14.3
	College or university completed	106	21.3
Occupational status	Government employee	109	21.9
	Retired	101	20.3
	Housewife	94	18.9
	Daily labourer	27	5.4
	Merchant	82	16.5
	Farmer	84	16.9
Residence	Urban	388	78.1
	Rural	109	21.9
Average family income	500–1000 ETB	63	12.7
	1001–2000 ETB	136	27.4
	2001–3000 ETB	88	17.7
	> 3000 ETB	210	42.3

Abbreviation: ETB = Ethiopian Birr.

### 3.2 The Magnitude of Dietary Nonadherence Among Individuals With DM

In this study, the level of dietary nonadherence among individuals with DM was 41.6% (95% CI: 37.4–46.0). On the perceived dietary adherence questionnaire, the highest mean score was obtained for the question “On how many of the last 7 days did you eat foods that contained or were prepared with vegetable oil, butter and sesame/nug oil?” and the lowest mean score was obtained for the question “On how many of the last 7 days did you eat fish or other foods high in omega‐3 fats?” (Table 2).

**Table 2:** Perceived dietary adherence questionnaire score of participants.

Variables/items	Mean ± SD
Dietary adherence	
Following a healthful eating plan	2.090 ± 2.090
Eating five or more servings of fruits and vegetables	1.63 ± 1.398
Eating CHO‐containing food with a low glycemic index	3.35 ± 1.871
Eating foods high in sugar, like rice and potatoes	1.57 ± 1.458
Eating foods high in fibre, like oatmeal and cereal	2.99 ± 2.178
Spacing CHO evenly throughout the day	0.90 ± 1.418
Eating fish or other foods high in omega‐3 fats	0.78 ± 1.188
Eating foods containing or prepared with vegetable oil	5.97 ± 1.815
Eating foods high in fat	2.04 ± 1.822
Overall adherence (*n*, %)	
Good	290 (58.4%)
Poor	207 (41.6%)

### 3.3 Behavioral, Psychosocial, and Clinical Characteristics

The mean duration of diabetes among participants was 8.1 years (±5.592). Of the 497 participants, 39.6% reported a lower level of physical activity in the last 7 days. Regarding social support, 43.9% of the participants reported receiving moderate support from their family, friends, and neighbors. Around 84.5% of the respondents had regular follow‐ups, and 35.8% of the participants received nutritional education during their follow‐up. In terms of glycemic control, more than two‐fifths (41.6%) of the participants had poor glycemic control. Additionally, out of 496 participants, nearly half (47.5%) have a healthy weight. Regarding waist circumference, three‐quarters of the participants (60.4%) had above‐normal waist circumference. Nearly three‐fifths of the participants (58.6%) had an above‐normal waist‐to‐hip ratio (Table 3).

**Table 3:** Behavioral, psychosocial, and clinical characteristics of participants.

Variables	Response category	Frequency (*n* = 497)	Per cent (100%)
Level of physical activity	High	114	22.9
	Moderate	186	37.4
	Low	197	39.6
Social support	Poor	151	30.4
	Moderate	218	43.9
	Strong	128	25.8
Type of DM	Type 1	104	20.9
	Type 2	393	79.1
Family history of HTN	No	304	61.2
	Yes	193	38.8
Family history of DM	No	271	54.5
	Yes	226	45.5
Regular follow‐up	No	75	15.1
	Yes	422	84.9
Nutritional education	No	178	35.8
	Yes	319	64.2
Comorbidity	No	322	64.8
	Yes	175	35.2
Diabetes‐related complications	No	396	79.7
	Yes	101	20.3
Average fasting blood glucose	Good	290	58.4
	Poor	207	41.6
Body mass index	Underweight	7	1.4
	Healthy weight	236	47.5
	Overweight	187	37.6
	Obese	67	13.5
Waist circumference	Normal	197	39.6
	Above normal	300	60.4
Waist‐to‐hip ratio	Normal	206	41.4
	Above normal	291	58.6

### 3.4 Factors Associated With Dietary Nonadherence Among Participants

In the bivariate logistic regression analysis, several variables showed a statistically significant association with dietary nonadherence at a *p* value < 0.25. These included participants' age, place of residence, marital status, educational status, occupational status, average family income, social support, comorbidities other than hypertension, and diabetes‐related complications. In the final multivariable logistic regression model, place of residence, marital status, occupational status, average family income, and social support remained significantly associated with dietary nonadherence at a *p* value ≤ 0.05 (Table 4).

**Table 4:** Bivariate and multivariate analysis results of participants.

Variables	Dietary adherence		COR (95%CI)	AOR (95% CI)	*p* value
	Good	Poor			
Age (years)					
≤ 50	94	80	1	1	
51–60	87	54	0.729 (0.464, 1.146)	0.793 (0.470, 1.338)	0.39
61–70	75	48	0.752 (0.470, 1.202)	0.969 (0.522, 1.797)	0.92
> 70	34	25	0.864 (0.476, 1.568)	1.191 (0.521, 2.721)	0.68
Marital status					
Married	213	143	1	1	
Single	20	27	2.011 (1.086, 3.722)	2.518 (1.248, 5.083)	**0.010**
Divorced	26	17	0.974 (0.510, 1.860)	0.701 (0.335, 1.468)	0.35
Widowed	31	20	0.961 (0.527, 1.752)	0.796 (0.397, 1.594)	0.52
Educational status					
Unable to read and write	49	61	2.421 (1.396, 4.197)	1.201 (0.454, 3.174)	0.71
Read and write	33	36	2.121 (1.141, 3.944)	1.316 (0.509, 3.407)	0.57
Primary school	52	36	1.346 (0.750, 2.416)	0.624 (0.257, 1.512)	0.30
Secondary school	38	15	0.768 (0.374, 1.577)	0.442 (0.173, 1.129)	0.09
Preparatory school	48	23	0.932 (0.492, 1.766)	0.665 (0.294, 1.504)	0.33
College or university completed	70	36	1	1	
Occupational status					
Government employee	74	35	1	1	
Retired	68	33	1.026 (0.575, 1.830)	1.088 (0.458, 2.580)	0.85
Housewife	44	50	2.403 (1.358, 4.252)	2.399 (0.981, 5.864)	0.06
Daily labourer	16	11	1.454 (0.611, 3.458)	1.259 (0.412, 3.849)	0.69
Merchant	55	27	1.038 (0.563, 1.913)	1.036 (0.437, 2.457)	0.94
Farmer	33	51	3.268 (1.803, 5.921)	5.032 (1.772, 14.293)	**0.002**
Residence					
Urban	238	150	1	1	
Rural	52	57	1.739 (1.134, 2.668)	0.307 (0.144, 0.658)	**0.002**
Average family income					
500–1000 ETB	19	44	4.632 (2.517, 8.522)	3.707 (1.623, 8.465)	**0.002**
1001–2000 ETB	74	62	1.676 (1.076, 2.609)	1.362 (0.731, 2.541)	0.33
2001–3000 ETB	57	31	1.088 (0.645, 1.835)	0.676 (0.354, 1.293)	0.24
> 3000 ETB	140	70	1	1	
Social support					
Poor	77	74	2.193 (1.339, 3.592)	2.535 (1.456, 4.412)	**0.001**
Moderate	124	94	1.730 (1.090, 2.746)	1.861 (1.118, 3.097)	**0.017**
Strong	89	39	1	1	
Presence of comorbidity					
Yes	112	63	0.695 (0.476, 1.016)	0.825 (0.523, 1.301)	0.41
No	178	144	1	1	
Presence of diabetes‐related complications					
Yes	67	34	0.654 (0.414, 1.034)	0.993 (0.581, 1.698)	0.98
No	223	173	1	1	

*Note:* Bold values indicate statistical significance at *p* < 0.05.

Single individuals with diabetes mellitus were 2.52 times more likely to exhibit dietary nonadherence compared to married individuals (AOR = 2.518; 95% CI: 1.248–5.083). Regarding occupational status, farmers with diabetes were 5.03 times more likely to have dietary nonadherence than government employees (AOR = 5.032; 95% CI: 1.772–14.293). Participants in urban areas were 69.3% less likely to have dietary nonadherence than those in rural areas (AOR = 0.307; 95% CI: 0.144–0.658). Those with an average monthly family income of 500–1000 ETB were 3.71 times more likely to have dietary nonadherence than participants with an income greater than 3000 ETB (AOR = 3.707; 95% CI: 1.623–8.465). Participants with poor social support were 2.54 times more likely to have dietary nonadherence than those with strong social support (AOR = 2.535; 95% CI: 1.456–4.412). Additionally, individuals with moderate social support had 1.86 times higher odds of dietary nonadherence than those with strong social support (AOR = 1.861; 95% CI: 1.118–3.097).

## 4 Discussion

We assessed dietary nonadherence and associated factors among individuals with diabetes mellitus attending treatment follow‐ups at the outpatient departments of comprehensive specialized hospitals in the Amhara region. This study's findings will help improve dietary adherence among participants by identifying key opportunities and challenges. Additionally, the results will provide valuable insights for health professionals and policymakers to address barriers to dietary adherence and develop practical dietary practice guidelines.

The level of dietary nonadherence in this study was 41.6% (95% CI: 37.4–46.0), comparable to the adherence level reported in a previous study conducted in Bahir Dar, Northwest Ethiopia [17]. However, this rate is lower than those reported in studies from Nepal [10] and various parts of Ethiopia, including Debre Tabor [15, 46], Dire Dawa [16], and Addis Abeba [11]. Conversely, it is higher than findings from studies conducted in Surat City, India [20], and Botswana [13]. This variation could be explained by the difference in social, economic, and cultural characteristics of the study participants and the difference in participants' understanding and perceptions of the role of diet in diabetes management [12].

In this study, single individuals with diabetes mellitus (DM) exhibited higher levels of dietary nonadherence compared to their married counterparts. This disparity may be attributed to the enhanced social support that married individuals often receive from their spouses and family members, which can positively influence adherence to dietary recommendations. Research indicates that spousal support significantly facilitates dietary adherence among individuals with type 2 diabetes [47]. Furthermore, studies have found that married individuals are more likely to adhere to nutritional recommendations than single individuals, potentially due to the social support provided by their spouses [48, 49].

Regarding occupational status, farmer individuals with DM had higher dietary nonadherence than governmental employees with DM. This finding is supported by studies in Yemen [21] and Ethiopia [50]. Furthermore, urban residents were less likely to have dietary nonadherence than rural residents. This finding aligns with studies conducted in Yemen [21] and Ethiopia [50]. However, some previous studies found no significant association between residency and dietary nonadherence among individuals with diabetes [9]. This finding could be explained by the fact that individuals in urban settings had greater access to information than those who resided in rural areas, who may not have received as much information. The data analysis by the residential group also revealed that most participants living in rural areas could not read and write (61.5%). Therefore, they could not exploit the information in the printed papers. In addition, rural areas may face challenges such as limited access to health education, lower literacy rates, and cultural practices that influence dietary habits. For instance, communal eating practices in rural communities may not accommodate individualized nutritional needs, potentially hindering adherence among individuals with diabetes.

Participants with a low average monthly family income had a higher level of dietary nonadherence than those with a high average monthly family income. This finding aligns with studies conducted in Peru [10] and Ethiopia [15, 50]. It may be attributed to the inability to afford the cost of recommended healthy foods.

In our study, a significant association was observed between the level of social support and dietary adherence among participants, consistent with findings from similar studies [12, 17, 49, 51, 52, 53]. This finding might be the result of depression. Depression may cause individuals to neglect their dietary habits, especially if they lack support from family or their broader social network. When people with diabetes do not receive support from their immediate social networks, they may start to exhibit behaviors like stress, isolation, frustration, anger, and guilt. These behavioral changes may cause individuals to disregard their diet [54, 55]. However, encouragement from loved ones can enhance motivation and inspire them to take positive steps toward better dietary adherence.

### 4.1 Limitations of the Study

Despite our best efforts to collect high‐quality data, this study has some limitations. First, social desirability bias may have influenced participants' responses, potentially leading to inaccurate findings. Additionally, because a cross‐sectional study design was used, it is impossible to establish causal relationships or determine the direction of associations between variables.

## 5 Conclusion and Recommendation

The present study observed a high nonadherence to dietary recommendations among individuals with diabetes mellitus. Factors such as marital status, place of residence, occupational status, average family income, and level of social support were significantly associated with dietary nonadherence. Therefore, health professionals should take a proactive role in identifying and addressing these barriers. In addition, healthcare policymakers should develop and implement practical dietary practice guidelines, particularly in settings lacking such resources.

The findings also highlight the importance of integrated interventions that strengthen social support networks and improve health information dissemination, including through printed materials such as posters. Further population‐based qualitative research is recommended to explore underlying and less visible factors affecting dietary practices. Moreover, future studies using longitudinal designs, portion size estimation, and repeated 24‐h dietary recalls are necessary to enhance understanding and improve dietary management of diabetes.

## Author Contributions

**Makda Abate Belew:** conceptualization, methodology, software, data curation, formal analysis, writing – review and editing, writing – original draft, supervision. **Rediet Akele Getu:** software, data curation, investigation, formal analysis, supervision, visualization, writing – review and editing. **Sewunnet Azezew Getahun:** conceptualization, methodology, software, data curation, supervision, visualization, writing – review and editing. **Belete Negese Gemeda:** software, data curation, investigation, validation, formal analysis, supervision, visualization, writing – review and editing. **Melese Wagaye Zergaw:** methodology, software, formal analysis, writing – review and editing, data curation. **Sewnet Getaye Workie:** conceptualization, methodology, software, data curation, supervision, writing – original draft, validation, writing – review and editing.

## Conflicts of Interest

The authors declare no conflicts of interest.

## Transparency Statement

The lead author, Makda Abate Belew, affirms that this manuscript is an honest, accurate, and transparent account of the study being reported; that no important aspects of the study have been omitted; and that any discrepancies from the study as planned (and, if relevant, registered) have been explained.

## Data Availability

The authors confirm that the data supporting this study's findings are available within the article and/or its supplementary materials.
